# Acromegaly discovered during a routine out-patient surgical procedure: a case report

**DOI:** 10.1186/s13256-017-1338-8

**Published:** 2017-06-24

**Authors:** Chukwudi O. Chiaghana, Julia M. Bauerfeind, Cheri A. Sulek, J. Christopher Goldstein, Caleb A. Awoniyi

**Affiliations:** 1 Department of Anesthesiology, University of Florida, 1600 SW Archer Road, Gainesville, FL 32608 USA; 2 Department of Anesthesiology, NF/SG Veterans Health System, Gainesville, FL 32608 USA

**Keywords:** Acromegaly, IGF-1, Growth hormone, Pituitary tumor, Endocrine tumor, Transsphenoidal, Difficult airway, Difficult intubation

## Abstract

**Background:**

Acromegaly is a rare syndrome in which there is unregulated hypersecretion of growth hormone. The anesthetic management of patients with this disorder is particularly challenging due to pre-existing cardiovascular and respiratory dysfunction, as well as recognized difficulties with airway management. Because of the insidious progression of the disease and the presence of nonspecific signs and symptoms, diagnosis is often made late when characteristic acromegalic features become apparent.

**Case presentation:**

We report the management of a 35-year-old African American man with previously undiagnosed acromegaly, who underwent a general anesthetic for same day surgery. Subtle physical features and difficult endotracheal intubation raised our suspicion for the diagnosis of acromegaly. Following an uncomplicated postoperative course he underwent workup for the disease, which was confirmed. In addition, brain magnetic resonance imaging showed a pituitary adenoma. A subsequent transsphenoidal hypophysectomy was performed successfully.

**Conclusions:**

This case underscores the notable absence of recognizing the clinical presentation of acromegaly in this patient by his primary care physician, and the value of thorough history taking, vigilance, and observation in making a new diagnosis that has the potential to alter a patient’s health care and mitigate impending morbidity and/or mortality.

## Background

Acromegaly is a rare clinical syndrome in which there is excessive secretion of growth hormone (GH); it is characterized by skeletal, soft, and connective tissue overgrowth. It is also often associated with systemic conditions such as hypertension, glucose intolerance, ischemic heart disease, pulmonary disorders, and malignancies. Because some of the clinical profiles associated with acromegaly are nonspecific and the manifestations of the disorder are insidious, several patients go undiagnosed for several years after the onset of initial signs and symptoms. While some studies estimate the prevalence of acromegaly as 40 to 125 per million [1], a recent cross-sectional epidemiological study estimated the prevalence at 1000 per million patients [2] suggesting under-diagnosis. Early diagnosis and prompt treatment of this disorder is vital as it carries up to 72% increase in all-cause mortality when compared to the general population [3]. Reportedly, internists diagnose 40% of acromegaly cases while the remainder is diagnosed by specialists seen for particular symptoms such as ophthalmologist for visual disturbances, oro-maxillofacial surgeons for bite disturbances, gynecologists for menstrual dysfunction and infertility, pulmonologists for obstructive sleep apnea, and rheumatologists for osteoarthritis [4]. In this paper, we report a case of unrecognized acromegaly suspected in a patient with characteristic clinical features and airway examination finding during a routine surgical procedure.

## Case presentation

A 35-year-old African American man with a history of hypertension, carpal tunnel syndrome, osteoarthritis of the knee, and glucose-6-phosphate deficiency (G6PD) was scheduled for removal of keloids found in the occipital region of his head. His medications include amlodipine, hydrochlorothiazide, lisinopril, and cholecalciferol. He was 1.85 m (73 inches) tall, weighed 134 kg with a body mass index of 39. An airway examination on the day of surgery showed a prominent mandible, limited mouth opening, macroglossia, positive prognathism, Mallampati IV classification, and thyromental distance (thyroid notch to the tip of the jaw with the head extended) >6 cm; a thyromental distance <6 cm serves as a predictor for difficult intubation. He also had other distinct skeletal features that included prominent supraciliary arches and nose bridge, as well as large hands and feet.

Following induction of general anesthesia with lidocaine, fentanyl, and propofol and muscle relaxation with succinylcholine, an elective Airtraq device was used for intubation with moderate difficulty. Following a second attempt, a 7.0 endotracheal tube was passed blindly into his trachea because of large immobile epiglottis that resulted in a grade IV indirect view. His surgical procedure was uneventful and he was extubated without difficulty. After full recovery in the post-anesthesia recovery unit, he was informed that he had a difficult airway and advised to inform all future anesthesia providers. In addition, because of the high index of suspicion for acromegaly he was educated about the possibility of having this disorder and was advised to follow up with his primary care provider for further evaluation.

**Fig. 1:**
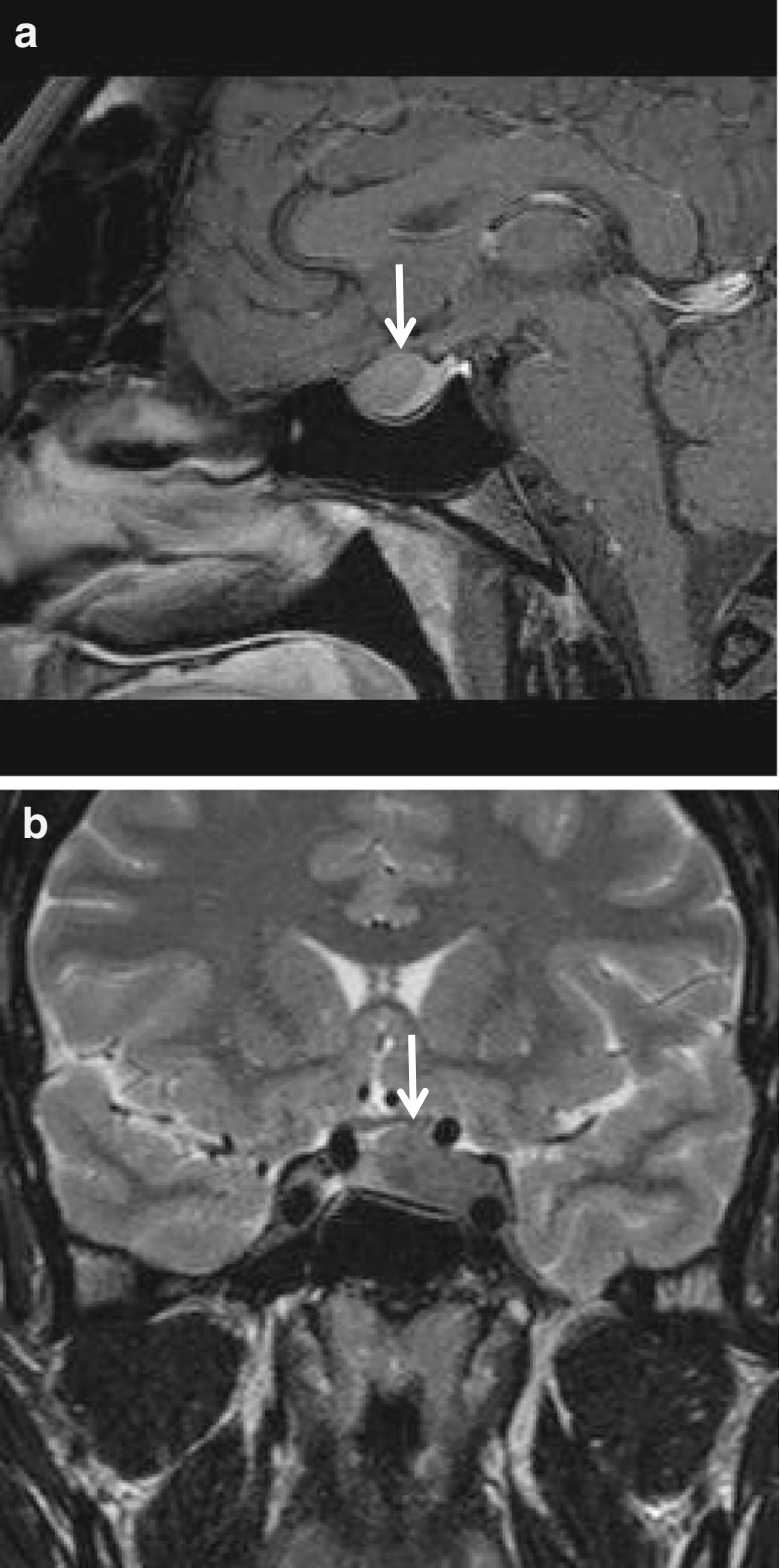
Magnetic resonance imaging of brain. **a** Sagittal section, and **b** coronal section; *arrow* indicates pituitary tumor

Endocrine tests performed subsequently included serum cortisol, adrenocorticotropic hormone, thyroid-stimulating hormone, prolactin, hemoglobin A1C, insulin-like growth factor 1 (IGF-1), GH, and parathyroid hormone levels. All of his biochemical markers were normal except for his serum IGF-1 and GH (Table 1) that were 700 ng/ml and 22.5 ng/ml, respectively: twofold greater than the reference normal upper limit. A transthoracic echocardiogram showed moderate left ventricular hypertrophy with preserved systolic left ventricular function. Magnetic resonance imaging of his brain showed a large mass arising from the sella and extending into the suprasellar cistern (19 × 12 × 20 mm), consistent with pituitary macroadenoma (Fig. 1).

**Table 1:** Serum hormone levels

Hormone	Pre-surgical	Post-surgical (14 days)	Post-surgical (21 days)	Normal levels
Prolactin	15.5 ng/ml	3.68 ng/ml	3.74 ng/ml	4–15.2 ng/ml
IGF-1	700 ug/ml	366 ug/ml	248 ug/ml	53–331 ug/ml
GH	22.5 ng/ml	0.4 ng/ml	0.6 ng/ml	<10 ng/ml

Abbreviations: *GH* growth hormone, *IGF-1* insulin-like growth factor 1

A neurosurgical evaluation was performed and he underwent a transsphenoidal resection of his pituitary tumor 3 months later. Given his known difficult airway, he was intubated using an awake oral fiberoptic technique. His perioperative period was uneventful. Pathological evaluation showed the resected pituitary adenoma to be focally reactive for prolactin and GH, and was negative for adrenocorticotropic hormone, thyroid-stimulating hormone, follicle-stimulating hormone, and luteinizing hormone. Serum laboratory tests performed 3 weeks postoperatively showed normalization of his prolactin, IGF-1, and GH levels (Table 1).

## Discussion

Acromegaly is a rare disorder with clinical manifestations that are primarily attributed to the systemic effect of sustained hypersecretion of GH. Complications associated with this disorder include cardiovascular dysfunction, respiratory compromise, malignancies, metabolic derangements, skeletal abnormalities, rheumatologic arthropathies, and neuropathies [5]. With these wide arrays of associated complications, it is not surprising that acromegaly carries increased morbidity and mortality. Some reports indicate a twofold to threefold increase in mortality when compared with the general population, with cardiovascular events accounting for the majority of mortality [6]. Other notable causes of death include respiratory complications and malignancies [7]. The increase in morbidity and mortality associated with acromegaly may be partly due to the insidious and progressive nature of the disease, which often leads to delayed diagnosis and/or treatment [8]. Therefore, it is imperative that clinicians from all specialties maintain a high index of suspicion on patients presenting with specific symptoms who have characteristic dysmorphic features of acromegaly.

Patients with acromegaly present an unusual challenge to anesthesiologists in the perioperative period. Multiple studies have documented a higher incidence of difficult intubation than in patients without acromegaly [9], with preoperative Mallampati III or IV identified as one of the predictive factors for difficult intubation [9, 10]. Mask ventilation is often difficult because of the dysmorphic facial anatomy in these patients. As a consequence of macroglossia, laryngeal mucosal and cartilage hypertrophy [11], visualization of vocal cords during direct laryngoscopy is often limited. Even when the vocal cords are visible, they may be hypertrophic, causing narrowed glottic opening and increased resistance to passage of endotracheal tube. With the rare opportunity of performing a thorough airway examination preoperatively and during laryngoscopy, anesthesiologists can therefore play a key role in early diagnosis of acromegaly, as was done in this patient. It is also imperative that a history of difficult intubations needs to be ascertained. Early detection will help circumvent not only the potential airway mishaps in subsequent surgeries in patients with undiagnosed acromegaly through proper planning but also help mitigate associated comorbidities that decrease quality of life and increase mortality.

When diagnosed, a pituitary adenoma is the primary cause of acromegaly. Depending on tumor size, peripheral visual disturbances may accompany the systemic manifestation of the disorder. Surgery, specifically transsphenoidal adenectomy, appears to be the mainstay therapeutic modality for most patients. When surgical intervention is not a good primary option such as in cases involving patients with advanced age, precarious medical status, or patient’s refusal, medical therapy, such as somatostatin analogs (octreotide and lanreotide), can provide adequate treatment modality by suppressing pituitary secretion of GH as well as hepatic inhibition of IGF-1 production [4, 12]. Following diagnosis and institution of appropriate treatment, it is also imperative that a comprehensive workup of all organ systems involved with this disorder be pursued.

## Conclusions

In summary, acromegaly can often remain undiagnosed until late in the disease process. Late diagnosis is due, in part, to the presence of nonspecific signs and symptoms (such as hypertension, diabetes, headache, obstructive sleep apnea), as well as slow progression of the characteristic features associated with the disease. Primary care physicians during routine visits and anesthesia personnel during preoperative evaluation should be cognizant of the clinical presentation of this disease. If not for a high index of suspicion of the disease, as observed in this case during a routine surgical procedure, it could remain undiagnosed until tumor resection becomes high risk leading to increased morbidity and mortality.
